# Intraductal patient‐derived xenografts of estrogen receptor α‐positive breast cancer recapitulate the histopathological spectrum and metastatic potential of human lesions

**DOI:** 10.1002/path.5200

**Published:** 2018-12-27

**Authors:** Maryse Fiche, Valentina Scabia, Patrick Aouad, Laura Battista, Assia Treboux, Athina Stravodimou, Khalil Zaman, RLS, Valerian Dormoy, Ayyakkannu Ayyanan, George Sflomos, Cathrin Brisken

**Affiliations:** ^1^ International Cancer Prevention Institute Epalinges Switzerland; ^2^ Swiss Institute for Experimental Cancer Research, School of Life Sciences Ecole Polytechnique Fédérale de Lausanne Lausanne Switzerland; ^3^ Centre Hospitalier Universitaire Vaudois University Hospital of Lausanne Lausanne Switzerland; ^4^ Réseau Lausannois du Sein (RLS) Lausanne Switzerland; ^5^ INSERM UMR_S1250 Université de Reims Champagne‐Ardenne (URCA) Reims France

**Keywords:** intraductal xenografts, luminal breast cancer, preclinical model, patient‐derived xenografts, ductal carcinoma *in situ*, micrometastasis

## Abstract

Estrogen receptor α‐positive (ER‐positive) or ‘luminal’ breast cancers were notoriously difficult to establish as patient‐derived xenografts (PDXs). We and others recently demonstrated that the microenvironment is critical for ER‐positive tumor cells; when grafted as single cells into milk ducts of NOD Scid gamma females, >90% of ER‐positive tumors can be established as xenografts and recapitulate many features of the human disease *in vivo*. This intraductal approach holds promise for personalized medicine, yet human and murine stroma are organized differently and this and other species specificities may limit the value of this model. Here, we analyzed 21 ER‐positive intraductal PDXs histopathologically. We found that intraductal PDXs vary in extent and define four histopathological patterns: flat, lobular, *in situ* and invasive, which occur in pure and combined forms. The intraductal PDXs replicate earlier stages of tumor development than their clinical counterparts. Micrometastases are already detected when lesions appear *in situ*. Tumor extent, histopathological patterns and micrometastatic load correlate with biological properties of their tumors of origin. Our findings add evidence to the validity of the intraductal model for *in vivo* studies of ER‐positive breast cancer and raise the intriguing possibility that tumor cell dissemination may occur earlier than currently thought. © 2018 The Authors. *The Journal of Pathology* published by John Wiley & Sons Ltd on behalf of Pathological Society of Great Britain and Ireland.

## Introduction

Breast cancer (BC) is a frequent disease worldwide 1. Over 75% of BCs express estrogen receptor (ER) in >1% of the tumor cells by immunohistochemistry (IHC) 2 and overlap with luminal A and B subtypes defined by global gene expression 3, 4 exhibiting low versus high proliferative indices and distant recurrence rates 5. Twenty percent of patients experience distant recurrence and cancer‐related death 6. Overtreatment of early disease and endocrine resistance are additional problems concerning this subgroup 7. A lack of preclinical models hampered progress in understanding the biology of luminal tumors and the development of new therapies. Genetically engineered mouse models mostly develop ER‐negative tumors; few ER‐positive BC cell lines grow *in vivo* requiring non‐physiological estrogen supplements 8. Patient‐derived xenografts (PDXs) are increasingly used but difficult to establish from ER‐positive tumors 8. We and others showed that the microenvironment is a major determinant of luminal BC cells and that take rates increase dramatically when luminal BC cells are grafted to mouse milk ducts 9. They grow without estrogen supplementation, recapitulating many features of their clinical counterpart 9, 10. Yet, mammary stroma and endocrine milieu differ between women and mice. To assess the impact of the mouse host on the biology of the engrafted human cells, we analyzed 21 intraductal PDXs histopathologically.

## Materials and methods

The study was approved by the Commission cantonale d'éthique de la recherche sur l'être humain (CER*‐*VD 38/15); patients signed informed consent. Animal experiments were performed in accordance with protocol 1861.3 approved by Service de la Consommation et des Affaires Vétérinaires, Canton de Vaud, Switzerland. After inking of margins and macroscopic assessment, part of the tumor tissue was taken by the pathologist (MF), transported to the laboratory in DMEM/F12, mechanically and enzymatically dissociated to single cells, lentivirally transduced with luciferase‐Green Fluorescent Protein (GFP) and injected into teats of 10‐week‐old NOD Scid gamma females 9. *In vivo* growth was monitored biweekly by bioluminescence. Engrafted glands were dissected, fixed in buffered formalin for 2 h and paraffin embedded. Four micrometer sections were cut and numbers 1, 7 and 15 stained with hemalun/eosin. Staining for Alu elements unequivocally identified human cells when required. IHC was performed on Discovery Ventana ULTRA 9. Micrometastatic load was calculated as the percentage of bioluminescence‐positive organs of all organs collected.

## Results

**Figure 1:**
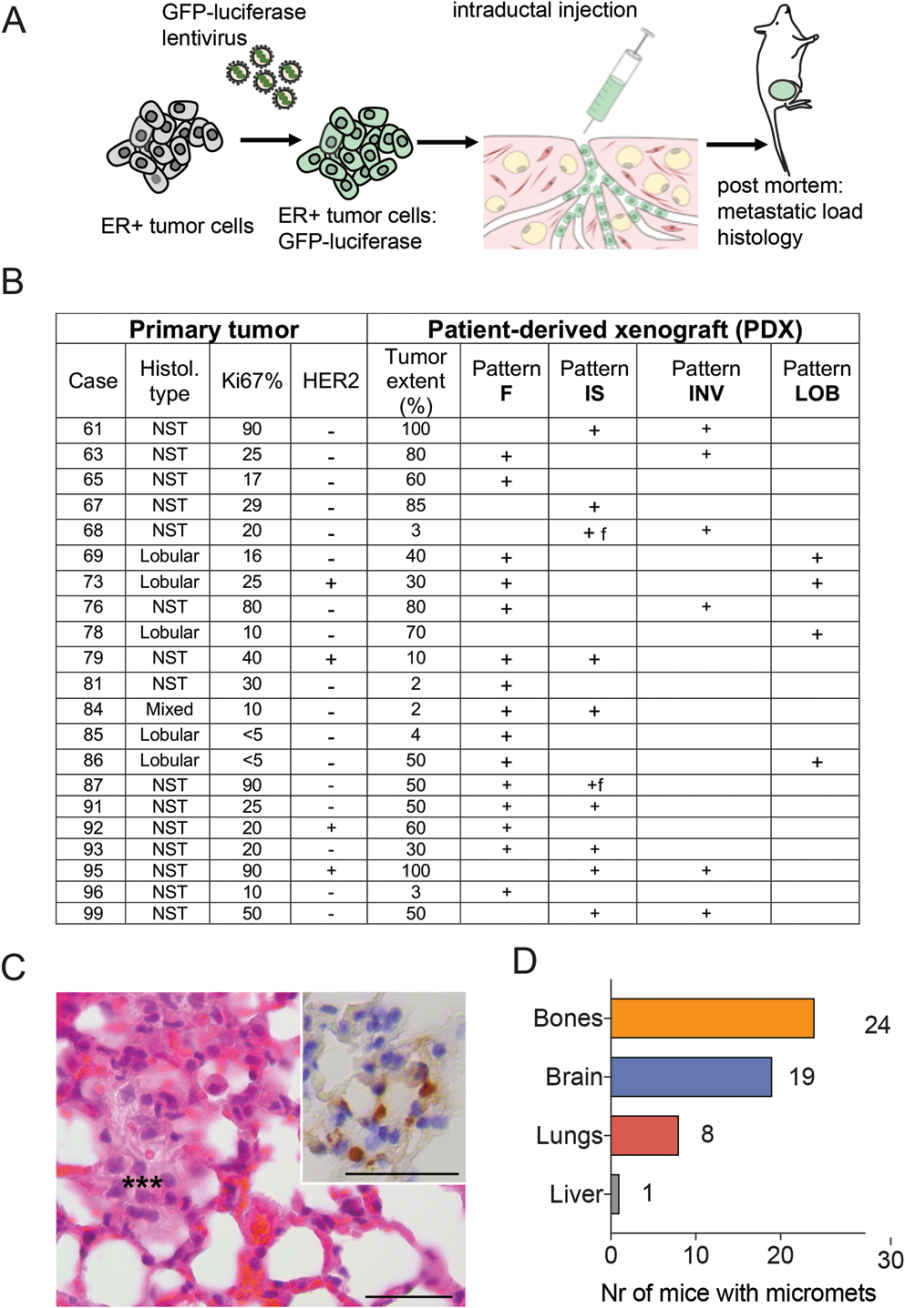
Intraductal ER‐positive BC PDXs. (A) Experimental scheme of PDX establishment and follow‐up. Primary tumors were dissociated to single cells, which were subsequently transduced with lentivirus encoding GFP and luciferase. The infected cells were injected intraductally in multiple glands and their *in vivo* growth monitored by bioluminescence. The mice were euthanized and the presence of metastases assessed by bioluminescence in brain, lungs, bones and liver. Analysis of spleen, intestine and other internal organs was negative. (B) Table reporting primary tumor and PDX characteristics. (C) Hemalum/eosin‐stained section of a micrometastasis*** in the lung, consisting of atypical cells with large nuclei. Inset: IHC staining for ER (brown) with Mayer's hematoxylin counterstain of an adjacent section revealing the ER‐positive tumor cells in the ER‐negative lung tissue. Scale bars = 35 μm. (D) Numbers of different organs (*n* = 88) in 31 xenografted mice bearing micrometastases.

Tumor cells from 21 patients were intraductally grafted to 88 mice in 220 glands after lentiviral transduction with luciferase‐GFP (Figure 1A,B, supplementary material, Table S1). Mice were sacrificed when radiance >10E8 so tumor cells are readily detectable. Micrometastases, consisting of <30 cells (Figure 1C), were detected in 90% of the 31 mice analyzed by *ex vivo* radiance measurements on various organs, revealing bones as the most frequent site of tumor cell seeding, followed by brain, lungs and liver (Figure 1D). Tumor extent assessed semiquantitatively on hemalun/eosin‐stained sections varied, with ≥70% of ducts distended by human cells in six of 21 PDXs (see supplementary material, Figure S1A) and tumor cell foci occupying 20–60% or <5% of the ductal tree in 10 and 5 PDXs, respectively (see supplementary material, Figure S1B,C).

Although the distribution of ER‐positive and progesterone receptor (PgR)‐positive indices in primary tumors and PDXs were similar (see supplementary material, Table S1 and Figure S2A,B), they differed by >10% in five and 12 pairs, respectively, consistent with higher inter‐ and intratumor variability of PgR staining in clinical samples (see supplementary material, Figure S2C,D). HER2 status was IHC 3+ in two of three PDXs corresponding to IHC 3+ primary tumors, including one confirmed by FISH. A primary tumor with an IHC 2+ score and focal gene amplification was negative in the PDX, suggesting clonal outgrowth.

**Figure 2:**
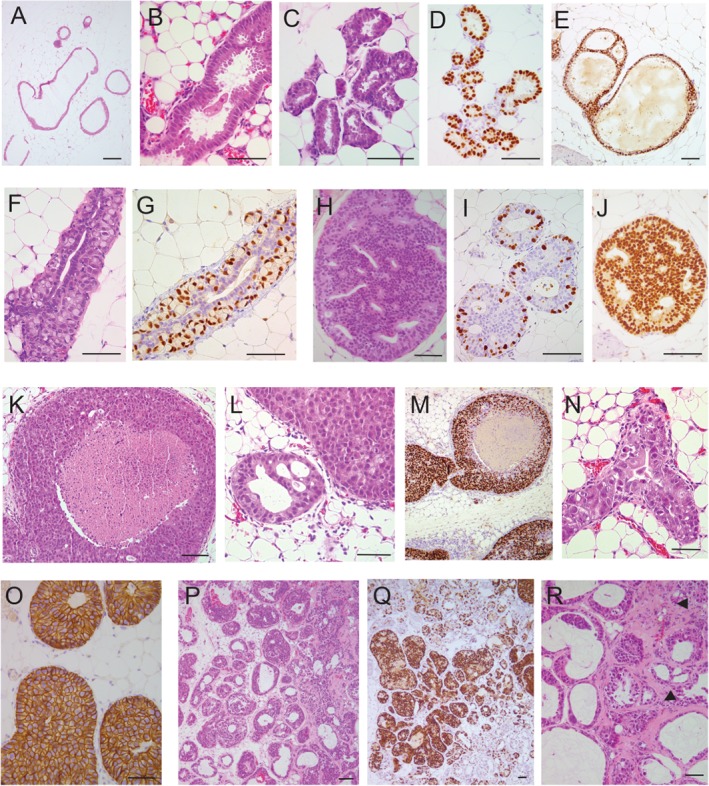
Intraductal PDXs exhibit four morphological patterns by hemalun/eosin staining and IHC. (A–E) Flat (F) pattern. (A–C) Human tumor cells cover the wall of variably dilated ducts forming a monolayer of large cylindrical cells with small nuclei, eosinophilic cytoplasm and apical cytoplasmic ‘snouts’ reminiscent of columnar cell changes and flat epithelial atypia in the human breast. (D and E) ER (D) and PgR (E) are strongly and diffusely expressed by human cells. (E) Focally, transition between the F pattern and cellular bridges similar to human atypical ductal hyperplasia is seen. (F and G) Lobular (LOB) pattern. (F) The mouse ductal epithelium is replaced by human cells harboring two associated phenotypes: (1) large cells with a ‘signet ring cell’ appearance (a large cytoplasmic vacuole displaces an enlarged nucleus toward the periphery of the cell) grow within the ductal wall in a ‘pagetoid’ manner; (2) smaller cohesive cubo‐cylindrical cells lining the ductal lumen. (G) ER is expressed in most large cells and in a few cylindrical cells. (H–O) *In situ* (IS) pattern. Human tumor cells fill the ductal lumen. (H–J) Some IS PDXs exhibit a cribriform architectural pattern, mild nuclear pleomorphism, low proliferation index (Ki67) (I) and diffuse strong ER expression (J). (K–O) One PDX exhibits a solid growth with comedonecrosis (K), marked nuclear pleomorphism (L and N), high proliferation index (Ki67) (M) and HER2 overexpression (O). (P–R) Invasive pattern (INV). Tumor cells diffusely expressing ER fill the dilated mouse ductal tree (P and Q) and grow outside the ducts in small clusters surrounded by collagen (P, R arrows). Scale bars = 100 μm.

The PDXs showed four distinct architectural patterns reminiscent of human breast disease: flat (F), lobular (LOB), *in situ* (IS) and invasive (INV) in pure form or mixed. The F pattern (Figure 2A–E) resembled columnar cell changes and flat epithelial atypia, an early premalignant alteration 11, 12, showing a monolayer lining of variably dilated mouse ducts (Figure 2A) with large columnar cells, mild nuclear pleomorphism and abundant eosinophilic cytoplasm forming apical ‘snouts’ (Figure 2B). It was associated with foci of intraductal proliferation of low nuclear grade tumor cells in a cribriform pattern, similar to atypical ductal hyperplasia (Figure 2E) 12. Strong and diffuse ER (Figure 2D) and PgR expression (Figure 2E) were observed. Together this pattern was interpreted as early ‘colonization’ of murine ducts by human tumor cells.

The LOB pattern was characterized by tumor cell growth within the ductal walls, like pagetoid spread of lobular carcinoma *in situ* (LCIS) (Figure 2F,G, supplementary material, Figure S3) 12, 13, associated with intracellular clear, mucin‐like vacuoles bestowing a signet‐ring cell‐like appearance on the tumor cells (Figure 2F). Interestingly, four of five lobular PDXs corresponded to this pattern; no typical LCIS was observed, instead four of them exhibited the F pattern. In the human breast, columnar cell changes and lobular neoplasia are frequently associated and considered as ‘low grade’ precursor lesions of both ductal and lobular subtypes 14, 15. The only case of pleomorphic lobular carcinoma showed LOB and F patterns with a 20% proliferative fraction (Figure 1B).

The IS pattern showed varying degrees of filling of the ductal lumen by tumor cells (Figure 2H–O) and resembled ductal carcinoma *in situ* (DCIS). It encompassed combinations of different degrees of nuclear pleomorphism and architectural patterns, like cribriform (Figure 2H–J) or solid with or without comedonecrosis (Figure 2K–O) 12. An ER‐positive HER2‐positive PDX showed large tumor extent, high nuclear pleomorphism, comedo architecture, high proliferation rate (Figure 2M) and HER2 overexpression (Figure 2O).

Although the tumor cells were derived from invasive primary tumors, only one‐third of the PDXs had an INV pattern, with tumor cells detected outside the host ducts, either isolated or in small clusters (Figure 2P–R). Thus, intraductal xenografts replicate earlier stages than the original primary tumor; nevertheless, micrometastases were present in 90% of the mice and in all the 16 analyzed PDXs (see supplementary material, Table S1).

**Figure 3:**
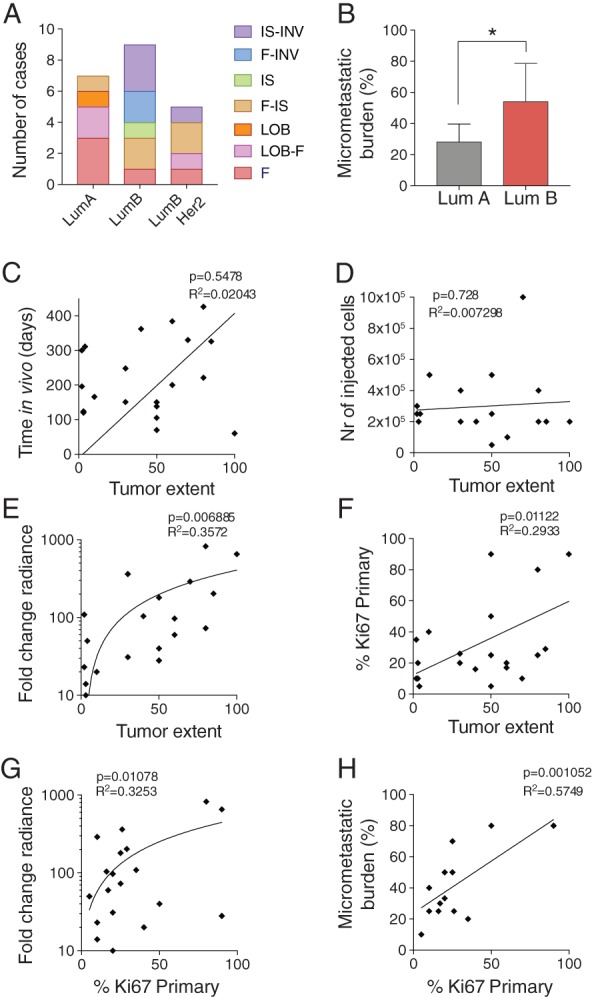
Characterization of *in vivo* growth. (A) Distribution of histological patterns within each subtype. (B) Micrometastatic burden expressed as percentage of positive organs in each recipient in luminal A‐ (LumA) versus luminal B‐ (LumB) derived PDXs. (C–F) Pearson correlation of tumor extent against (C) time of PDX growth *in vivo*, expressed in days after injection, (D) initial number of cells injected per gland, (E) *in vivo* cell growth expressed as fold‐change radiance relative to the first day of measurement (log10) and (F) Ki67 index of the primary tumor. (G and H) Pearson correlation of primary tumor Ki67% with (G) *in vivo* monitored growth and with (H) micrometastatic burden the percentage of organs affected by metastases within each case as determined by bioluminescence.

Next, we assessed how the biological features of the PDXs related to the prognostic characteristics of their clinical counterparts. According to a 20% Ki67 IHC cut‐off based on clinical practice 16, the primary tumor series comprised seven luminal A‐like (Ki67 < 20%) and 14 luminal B‐like (Ki67 ≥ 20%) cases; four of the latter were HER2‐positive. In luminal A‐like cases, the F pattern was observed in six of seven PDXs, pure in three cases, mixed with IS in one and with the LOB pattern in two cases. No INV pattern was found and the IS pattern was only found in one case (Figures 1B and 3A). Nine of 14 luminal B‐like cases, all of no special type (NST) (12) showed the F pattern, only 2 of them as the only pattern (Figure 3A). The INV pattern was detected in six of 14; IS and INV patterns were combined in four of 14. Thus, the F pattern tended to be present in luminal A‐like BC‐derived PDXs. IS and INV patterns on the contrary were observed, with or without the associated F pattern, in PDXs obtained from the more aggressive luminal B‐like subtype. Micrometastatic burden was higher in mice engrafted with luminal B‐like than luminal A‐like primary tumors (Figure 3B).

Tumor extent did not correlate with the time the grafted cells spent in the hosts (Figure 3C) nor with the number of cells injected (Figure 3D) but with *in vivo* growth rates (Figure 3E) and Ki67 index in the primary tumor (Figure 3F). The proliferative indices in primary tumors also correlated with *in vivo* growth rates (Figure 3G) and micrometastatic load (Figure 3H). Thus, tumor extent relates to primary tumor biology rather than to engraftment modalities; growth rates and micrometastatic burden reflect Ki67 index and hence patient prognosis.

## Discussion

The intraductal PDXs of ER‐positive BCs in this study reproduce the spectrum of ER‐positive DCIS with different architectures: solid, comedo and cribriform, combined with various degrees of nuclear pleomorphism 12, 13, 17. Several precursor lesions, such as flat epithelial atypia, atypical ductal hyperplasia, pagetoid growth of LCIS, were observed, whereas typical aspects of LCIS were not. Invasive cancer occurred only as small foci and no true invasive lobular carcinomas or other special subtypes were observed in the 21 PDXs under study.

Hence, most histopathological features are tumor cell‐intrinsic and not determined by stroma or systemic factors, which differ between humans and mice. The observation that two‐thirds of the PDXs derived from invasive cancers represent earlier lesions suggests that engrafted cells re‐start growth intraductally, recapitulating different steps with a prolonged stage of non‐invasive growth as they probably did in the patient's breast years before diagnosis. This is important to consider when using the model in personalized medicine 9.

Although the typical F, LOB and IS patterns are readily identified, some morphological aspects of ER‐positive intraductal PDX are more difficult to classify. This concerns dispersed foci of human cells lining the wall of small mouse ducts, with neither apical snouts nor cystic duct dilation. We classified these as F but speculate that they will evolve into a different pattern. Although most (15/21) PDXs had some F pattern, it tended to be pure in luminal A‐derived PDXs, whereas IS and INV patterns were detected when luminal B cells were engrafted. Furthermore, proliferative indices of primary tumors were maintained in the PDXs. Thus, the heterogeneity of luminal tumors was preserved. This is especially important regarding the clinical problem of overtreatment; the model may help identify tumors with low aggressiveness and/or a high level of responsiveness to endocrine therapy.

## Author contributions statement

AT, AS, KZ and the RLS selected patients for participation in the study, obtained informed consent and participated in the planning of tumor collection with LB. VS, PA, AA, VD and GS performed experiments and analyzed data. MF took tumor samples. MF and GS analyzed PDX sections. MF and CB wrote the manuscript. All authors were involved in writing the paper and had final approval of the submitted and published versions.

**SUPPLEMENTARY MATERIAL ONLINE**

**Figure S1.** Different tumor extents in PDXs

**Figure S2.** Marker expression

**Figure S3.** Human cells colonize mouse milk duct

**Table S1.** Characterization of primary tumors (PTs) and PDXs

## Supporting information


**Figure S1.** Different tumor extents in PDXs. (A) High tumor extent: Intraductally injected human tumor cells occupy over 95% of the mouse mammary ductal tree (H&E, × 4). (B) Moderate tumor extent: human tumor cells occupy between 10% and 50% of the mouse ductal tree (Alu staining, ×x 4). (C) Low tumor extent: human tumor cells form small foci occupying less than 5% of the mouse mammary ductal tree (ALU staining,). Scale bars: 800 μm
**Figure S2.** Marker expression. (A, C) Distribution of ER and PgR expression in PTs and PDXs, (B, D) Matched ER and PgR expression indices of PT and of the intraductal PDXs
**Figure S3.** Human cells colonize mouse milk duct. (A, B) Alu stained section of mouse mammary gland intraductally engrafted with cells derived from a lobular carcinoma. Scale bars: 100 μm and 50 μm

**Table S1.** Characterization of primary tumors (PTs) and PDXs
