# Supplementary material for: Intraductal patient‐derived xenografts of estrogen receptor α‐positive breast cancer recapitulate the histopathological spectrum and metastatic potential of human lesions
Source: J Pathol. 2018 Dec 27;247(3):287–92. doi: 10.1002/path.5200 (PMC6590246; doi:10.1002/path.5200)
Supplement: Supplementary file 1 — Figure S1. Different tumor extents in PDXs. (A) High tumor extent: Intraductally injected human tumor cells occupy over 95% of the mouse mammary ductal tree (H&E, × 4). (B) Moderate tumor extent: human tumor cells occupy between 10% and 50% of the mouse ductal tree (Alu staining, ×x 4). (C) Low tumor extent: human tumor cells form small foci occupying less than 5% of the mouse mammary ductal tree (ALU staining,). Scale bars: 800 μm Figure S2. Marker expression. (A, C) Distribution of ER and PgR expression in PTs and PDXs, (B, D) Matched ER and PgR expression indices of PT and of the intraductal PDXs Figure S3. Human cells colonize mouse milk duct. (A, B) Alu stained section of mouse mammary gland intraductally engrafted with cells derived from a lobular carcinoma. Scale bars: 100 μm and 50 μm [file PATH-247-287-s001.pdf]

A B C

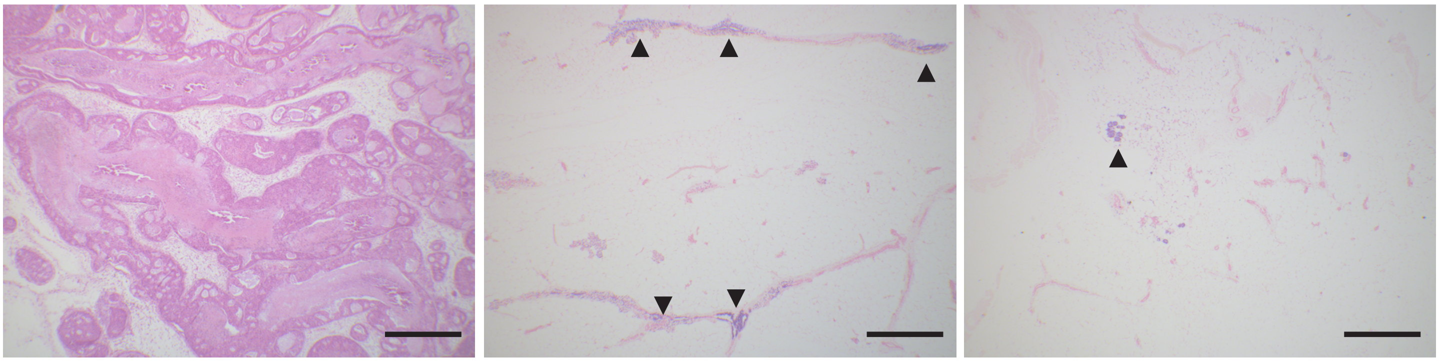

Figure S1. Different tumor extents in PDXs.
(A) High tumor extent: Intraductally injected human tumor cells occupy over 95% of the mouse mammary ductal tree (H&E, x 4). (B) Moderate tumor extent: human tumor cells occupy between 10% and 50% of the mouse ductal tree (Alu staining, x 4). (C) Low tumor extent: human tumor cells form small foci occupying less than 5% of the mouse mammary ductal tree (ALU staining,). Scale bars: 800 µm.

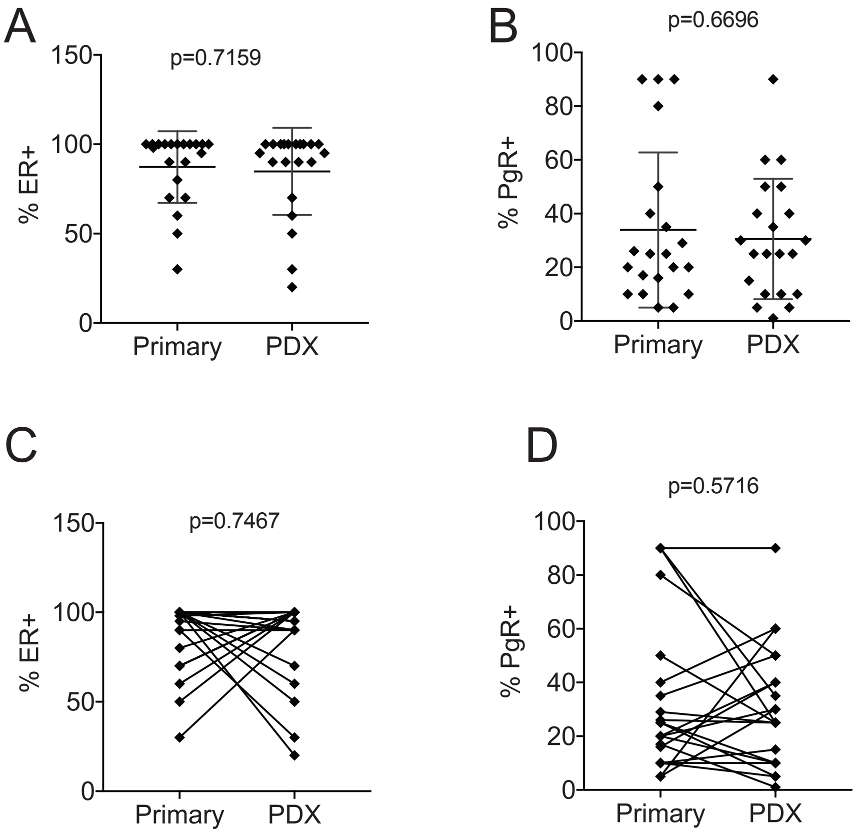

Figure S2. Marker expression.
(A, C) Distribution of ER and PgR expression in PTs and PDXs, (B, D) Matched ER and PgR expression indices of PT and of the intraductal PDXs

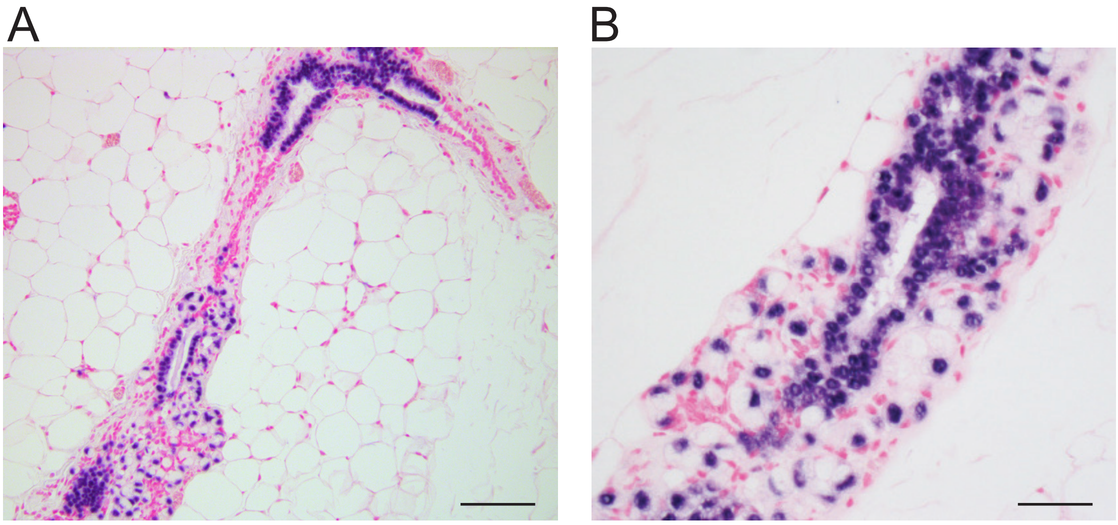

Figure S3. Human cells colonize mouse milk duct.
(A, B) Alu stained section of mouse mammary gland intraductally engrafted with cells derived from a lobular carcinoma. Scale bars: 100 μm and 50 μm.
